# Bystander Host Cell Killing Effects of *Clostridium perfringens* Enterotoxin

**DOI:** 10.1128/mBio.02015-16

**Published:** 2016-12-13

**Authors:** Archana Shrestha, Matthew R. Hendricks, Jennifer M. Bomberger, Bruce A. McClane

**Affiliations:** Department of Microbiology and Molecular Genetics, University of Pittsburgh School of Medicine, Pittsburgh, Pennsylvania, USA

## Abstract

*Clostridium perfringens* enterotoxin (CPE) binds to claudin receptors, e.g., claudin-4, and then forms a pore that triggers cell death. Pure cultures of host cells that do not express claudin receptors, e.g., fibroblasts, are unaffected by pathophysiologically relevant CPE concentrations *in vitro*. However, both CPE-insensitive and CPE-sensitive host cells are present *in vivo*. Therefore, this study tested whether CPE treatment might affect fibroblasts when cocultured with CPE-sensitive claudin-4 fibroblast transfectants or Caco-2 cells. Under these conditions, immunofluorescence microscopy detected increased death of fibroblasts. This cytotoxic effect involved release of a toxic factor from the dying CPE-sensitive cells, since it could be reproduced using culture supernatants from CPE-treated sensitive cells. Supernatants from CPE-treated sensitive cells, particularly Caco-2 cells, were found to contain high levels of membrane vesicles, often containing a CPE species. However, most cytotoxic activity remained in those supernatants even after membrane vesicle depletion, and CPE was not detected in fibroblasts treated with supernatants from CPE-treated sensitive cells. Instead, characterization studies suggest that a major cytotoxic factor present in supernatants from CPE-treated sensitive cells may be a 10- to 30-kDa host serine protease or require the action of that host serine protease. Induction of caspase-3-mediated apoptosis was found to be important for triggering release of the cytotoxic factor(s) from CPE-treated sensitive host cells. Furthermore, the cytotoxic factor(s) in these supernatants was shown to induce a caspase-3-mediated killing of fibroblasts. This bystander killing effect due to release of cytotoxic factors from CPE-treated sensitive cells could contribute to CPE-mediated disease.

## IMPORTANCE

In susceptible host cells, *Clostridium perfringens* enterotoxin (CPE) binds to claudin receptors and then forms pores that result in cell death. Using cocultures of CPE receptor-expressing sensitive cells mixed with CPE-insensitive cells lacking receptors for this toxin, the current study determined that CPE-treated sensitive cells release soluble cytotoxic factors, one of which may be a 10- to 30-kDa serine protease, to cause apoptotic death of cells that are themselves CPE insensitive. These findings suggest a novel bystander killing mechanism by which a pore-forming toxin may extend its damage to affect cells not directly responsive to that toxin. If confirmed to occur *in vivo* by future studies, this bystander killing effect may have significance during CPE-mediated disease and could impact the translational use of CPE for purposes such as cancer therapy.

## INTRODUCTION

*Clostridium perfringens* enterotoxin (CPE) is a 35-kDa single polypeptide that lacks significant primary amino acid sequence homology with other toxins (1), but structurally it belongs to the aerolysin pore-forming toxin family (2–4). CPE causes the gastrointestinal symptoms of *C. perfringens* type A food poisoning, which is the second most common bacterial foodborne illness (1, 5, 6) in the United States, where it affects ~1 million people/year (7). Similarly, CPE production is necessary for *C. perfringens* type A strains to cause ~5 to 10% of all human nonfoodborne gastrointestinal disease cases (6, 8). This toxin may also contribute to some human enteritis necroticans cases caused by CPE-producing type C strains of *C. perfringens* (9).

CPE action begins when this toxin binds to claudin receptors on host cells. Claudins, a large family of proteins that typically have a mass of ~20 to 27 kDa, are important mammalian tight junction components (10). Some claudins (e.g., claudin-1) bind CPE poorly or not at all, while other claudins are receptors with strong (e.g., claudin-3 or -4) or moderate (e.g., claudin-8 or -14) CPE binding affinity (11–15).

Once bound to a claudin receptor, CPE becomes sequestered in an ~90-kDa small complex on the host cell surface (16). Those small CPE complexes then rapidly oligomerize into an ~450-kDa prepore containing ~6 CPE molecules (17, 18, 19). When each CPE in the prepore extends a β-hairpin loop, this results in formation of a β-barrel pore in plasma membranes (20). This pore (named CH-1 [19]) allows rapid Ca^2+^ influx into the host cell cytoplasm (21–23). At high CPE doses, a massive calcium influx causes strong calpain activation and host cells die via a form of necrosis known as oncosis (23, 24). At lower CPE doses, where there is less calcium influx and calpain activation, a classical caspase-3/7-mediated apoptosis develops (23, 24). Enterocyte cell death leads to intestinal damage and increased fluid and ion secretion (25–27).

Pure cultures of mammalian cells that do not produce claudin receptors are insensitive to pathophysiologically relevant CPE concentrations (15). However, both CPE-sensitive cells and CPE-insensitive cells are present *in vivo*. Therefore, this study evaluated whether CPE might affect insensitive cells in coculture with sensitive cells.

## RESULTS

### CPE treatment affects the viability of parent cells cocultured with claudin-4 transfectant cells.

**FIG 1:**
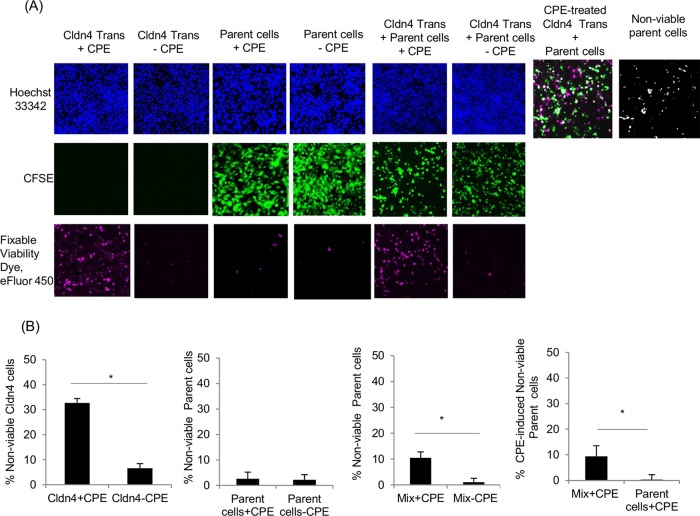
Microscopic evaluation of CPE cytotoxic effects on parent cells cocultured with claudin-4 transfectant cells. (A) Confocal fluorescence microscopy of claudin-4 transfectant (Cldn4 Trans) cells, parent cells, or a 1:1 mixture of claudin-4 transfectant cells and parent cells that were treated with 0.5 μg ml^−1^ CPE for 1 h (+ CPE) or were not treated with CPE (− CPE). Parent cells were stained with CFSE (green) prior to this CPE treatment. Dead cells were identified using fixable viability dye eFluor 450 (dead cells stain pink). An overlay (rightmost two panels) of nonviable cells in the mixed culture of parent cells and claudin-4 transfectant cells produced white spots indicative of dead parent cells. The total number of cells present in each culture was determined by staining with Hoeschst 33342. Results shown are representative of three experimental repetitions. (B) Quantitative analysis of cytotoxicity results in panel A. Nonviable cells were counted microscopically in both pure and mixed cultures, and the percentage of cell death was calculated in the three left panels. In the rightmost panel, CPE-induced death (total death minus background death) is compared between parent cells in mixed culture versus pure culture. Results are the means of three independent experiments. Error bars represent the standard errors of the means. Values that are significantly different (*P* < 0.05) are indicated by a bar and asterisk.

Before testing whether CPE treatment might damage naturally CPE-insensitive parent cells in coculture with CPE-sensitive cells, control experiments first demonstrated that (i) 5- (and 6-)-carboxyfluorescein diacetate succinimidyl ester (CFSE) staining did not itself affect the viability of parent cells and (ii) pure cultures of those CFSE-stained parent cells remained insensitive to CPE (Fig. 1A and B).

In contrast, CFSE-stained parent cells lost significant viability when treated with CPE in coculture with CPE-sensitive claudin-4 transfectant cells (Fig. 1A and B). After subtracting the nonviable parent cell background (no CPE treatment) always present in coculture with claudin-4 transfectants, ~10% of parent cells were rendered nonviable by a 0.5-μg ml^−1^ CPE dose in this coculture. This result was significantly higher than the <1% of nonviable cells detected after similar CPE treatment of a pure culture of parent cells.

### Supernatants collected from CPE-treated sensitive cell cultures reduce parent cell viability.

**FIG 2:**
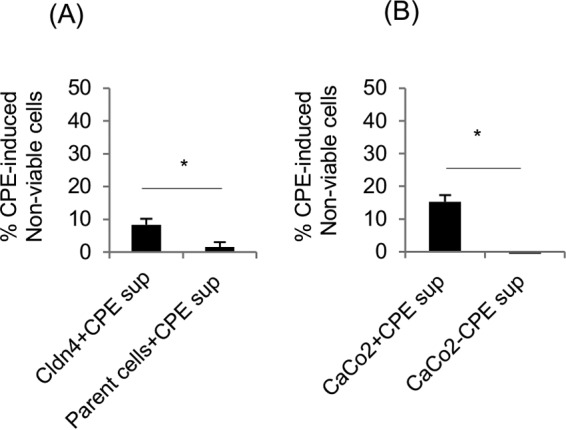
Culture supernatants from CPE-treated sensitive cells induce cytotoxicity in pure cultures of parent cells. (A) Cytotoxic effects on parent cells of supernatants collected from claudin-4 transfectant cells (Cldn4+CPE sup) or supernatants collected from parent cells (Parent cells+CPE sup) that had been treated for 1 h with 0.5 μg ml^−1^ of CPE, as measured by fluorescence microscopy. (B) Cytotoxic effects on parent cells of supernatants collected from Caco-2 cells that had been treated for 1 h with 1 μg ml^−1^ of CPE (+CPE) or without CPE (-CPE), as measured by the MTT assay. Results shown are the means of three repetitions; error bars represent the standard errors of the means. Values that are significantly different (*P* < 0.05) are indicated by a bar and asterisk.

The CPE-induced parent cell cytotoxicity detected in Fig. 1 could involve a factor(s) released from CPE-treated claudin-4 transfectant cells. If this is the case, then pure cultures of parent cells should exhibit less viability when treated with supernatants collected from pure cultures of CPE-challenged claudin-4 transfectant cell cultures than when treated with supernatants collected from similarly CPE-challenged pure parent cell cultures. This hypothesis was verified (Fig. 2A) using the Fig. 1 microscopy approach and confirmed using 3-(4,5-dimethylthiazol-2-yl)-2,5-diphenyltetrazolium bromide (MTT) and lactate dehydrogenase (LDH) cytotoxicity assays (not shown). The release of cytotoxic factor(s) into culture supernatants is not restricted to CPE-sensitive claudin-4 transfectants, since this effect was also observed using supernatants from human CPE-treated Caco-2 cells, which are pathophysiologically relevant enterocyte-like cells (Fig. 2B).

**FIG 3:**
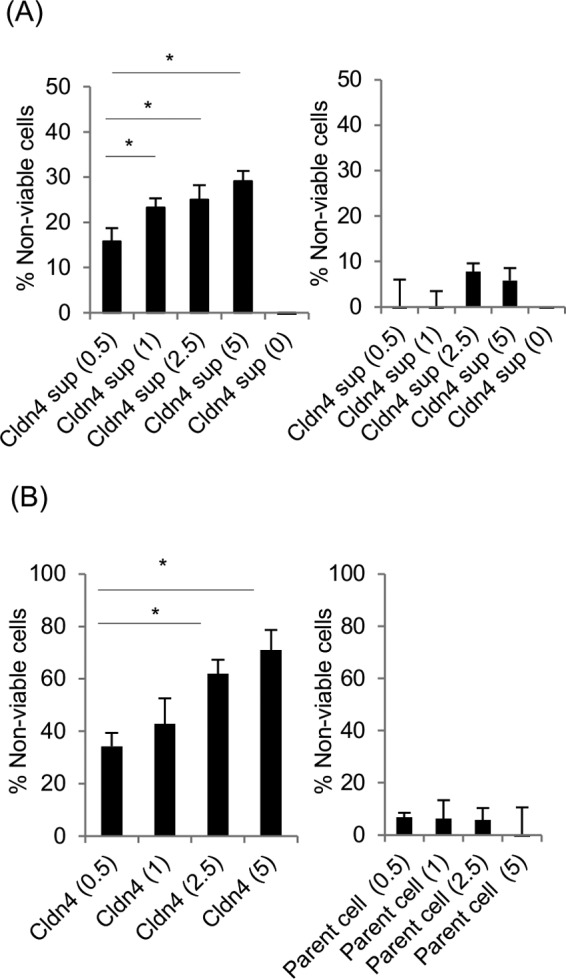
CPE dosage effects on culture supernatant cytotoxic activity. (A) Cytotoxic effects on parent cells of supernatants collected from claudin-4 transfectant cells. (Left) Cytotoxic effects of supernatants from claudin-4 transfectant cells treated with the indicated CPE dose (0, 0.5, 1.0, 2.5, or 5 μg ml^−1^ CPE). An MTT assay was performed to quantify cytotoxicity. All values were significantly higher (*P* < 0.05) from the preceding toxin dose except for the 1  μg ml^−1^ versus 2.5 μg ml^−1^ CPE doses. (Right) Cytotoxic effects on parent cells of supernatants collected from claudin-4 transfectant cells treated with buffer for 1 h at 37°C; CPE (0, 0.5, 1.0, 2.5 or 5 μg ml^−1^ CPE) was then added before the supernatants were applied to parent cells, and an MTT viability assay was performed. (B) Direct treatment of claudin-4 transfectant cells (left) or parent cells (right) for 1 h with CPE (0.5, 1.0, 2.5, or 5 μg ml^−1^). All values represent the means of three independent experiments. Error bars represent the standard errors (SE). Values that are significantly different (*P* < 0.05) are indicated by a bar and asterisk.

The Fig. 1 and 2 experiments used low CPE doses to minimize cell detachment (as necessary for microscopy analysis), so a CPE dose-response experiment was performed with an MTT assay (Fig. 3A, left panel). This analysis showed that CPE dosage affects the extent of parent cell cytotoxicity caused by supernatants collected from claudin-4 transfectant cells. This cytotoxicity was not a direct effect of high CPE doses on parent cell viability, since adding the same CPE doses to supernatants collected from control claudin-4 transfectant cell cultures (no CPE treatment) had no effect on parent cell viability (Fig. 3A, right panel). Last, while the cytotoxic effects of supernatants collected from CPE-treated claudin-4 transfectant cells on parent cells were significant, they were less than those observed in claudin-4 transfectant cells treated directly with comparable CPE amounts (Fig. 3A and B, left panels). As expected, parent cells treated directly with the same high CPE doses showed no altered viability (Fig. 3B, right panel).

### Initial characterization of release of cytotoxic factor(s).

**FIG 4:**
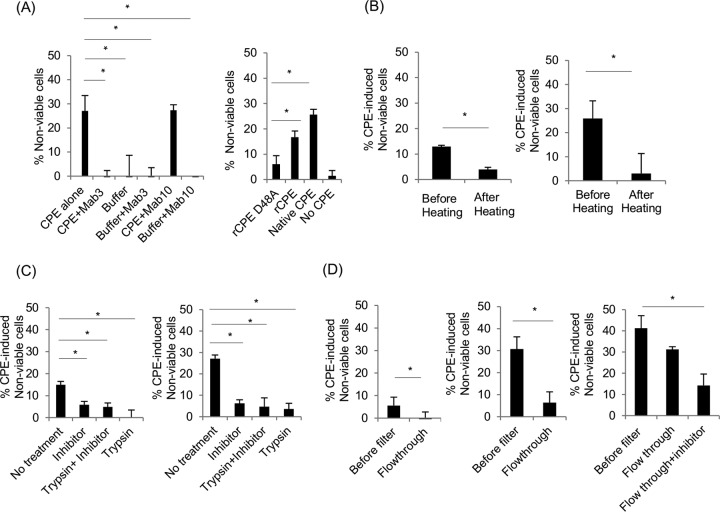
Characterization of release of cytotoxic factor(s) from CPE-treated sensitive cells**.** (A) Active CPE (1 μg ml^−1^) is needed for cytotoxic factor release from CPE-treated sensitive cells. (Left) Samples (*x* axis) included CPE alone, CPE with MAb3 antibody, CPE with MAb10 antibody, buffer alone, MAb3 alone, or MAb10 alone. The samples were preincubated for 1 h before being applied to claudin-4 transfectant cells for 1 h. Supernatants were collected from those cells, briefly centrifuged at 2,000 × *g*, and then applied to parent cells for 1 h before cytotoxicity testing using the MTT assay. (Right) rCPE-D48A, rCPE, or native CPE (1 μg ml^−1^) was added to claudin-4 transfectant cells for 1 h. Supernatants from those cultures were then applied to parent cells for 1 h, and an MTT assay was performed. (B to D) Sensitivity and size analyses of the cytotoxic factor(s) released from claudin-4 transfectant cells. Samples characterized included crude supernatants from claudin-4 transfectant cells treated for 1 h with 0.5 μg ml^−1^ of CPE (left graphs) or supernatant fractions that were collected from claudin-4 transfectant cells treated with 5 μg ml^−1^ and then depleted of membrane vesicles by centrifugation at 100,000 × *g* (right graphs for panels B and C and the middle graph for panel D). (B) Heat sensitivity. Cytotoxic activity remaining in crude supernatants or supernatants depleted of membrane vesicles before and after heating at 70°C for 10 min. (C) Protease sensitivity of cytotoxic factors in crude supernatants or supernatants depleted of membrane vesicles was assessed as follows: (i) supernatant alone (No treatment), (ii) supernatant treated with 200 μg trypsin inhibitor (Inhibitor), (iii) supernatant treated with 100 μg trypsin, followed by the addition of 200 μg trypsin inhibitor (Trypsin+Inhibitor), and (iv) supernatant treated with 100 μg trypsin (Trypsin). (D) Size of cytotoxic factor(s) in crude supernatants that were collected from CPE-treated claudin-4 transfectant cells and then depleted of membrane vesicles. Crude supernatants (left) or membrane-depleted supernatants (middle) were filtered through a 10,000-molecular-weight-cutoff Amicon filter. (Right) Cytotoxic activity of membrane-depleted supernatant filtered through a 30-kDa-cutoff filter, with or without the addition of 200 μg ml^−1^ trypsin inhibitor to the flowthrough. All samples in panels B to D were applied to parent cells for 1 h before an MTT assay was performed to determine cytotoxicity. Results shown in panels A to D are means from three independent experiments. Error bars represent the standard errors of the means. Values that are significantly different (*P* < 0.05) are indicated by a bar and asterisk.

To begin characterizing release of cytotoxic factor(s) by CPE-treated claudin-4 transfectant cells, we asked whether this effect requires CPE action. Preincubating the toxin with a CPE-neutralizing monoclonal antibody blocked cytotoxic factor release from claudin-4 transfectant cells (Fig. 4A, left panel); this effect was specific, since similar preincubation using a non-CPE-neutralizing monoclonal antibody did not affect cytotoxic factor release from claudin-4 transfectant cells. The importance of fully active CPE for inducing the release of cytotoxic activity from CPE-treated sensitive cells was confirmed when cytotoxic factor release from claudin-4 transfectant cells was detected (Fig. 4A, right panel) after treatment of those cells with native CPE or recombinant CPE (rCPE), but not after similar treatment with rCPE-D48A (rCPE with D48A change), which binds and forms a small complex but cannot form any CH-1 pores (26).

Additional characterization studies suggested the cytotoxic activity of supernatants collected from CPE-treated claudin-4 transfectant cell cultures may involve a heat-labile protein. The cytotoxic activity in those supernatants was reduced by heating (Fig. 4B, left panel) or trypsin treatment (Fig. 4C, left panel). Interestingly, supernatant cytotoxic activity was also impaired by the addition of trypsin inhibitor (Fig. 4C, left panel). The results of ultrafiltration experiments suggested that the cytotoxic factor(s) in crude supernatants has a molecular mass of >10 kDa (Fig. 4D, left panel).

### Evaluating whether CPE species are the supernatant cytotoxic factor affecting parent cells.

**FIG 5:**
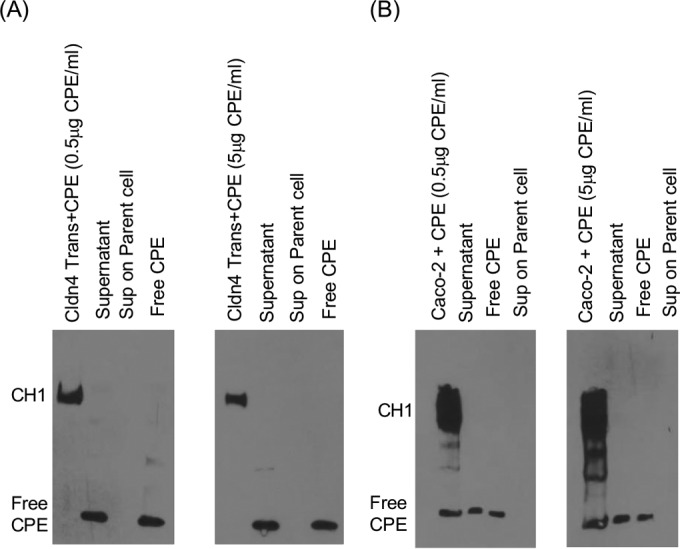
Presence of CPE species in supernatants of CPE-treated cells and in parent cells treated with those supernatants. (A and B) Confluent claudin-4 transfectant cells (A) or Caco-2 cells (B) were treated with 0.5 or 5.0 μg ml^−1^ of CPE for 1 h. The supernatants from those cultures were collected, and after the cells were washed, the CPE-treated cells were subjected to CPE Western blotting (leftmost lane of each blot). The supernatants from those CPE-treated cultures were centrifuged briefly at 2,000 × *g*. An aliquot of each centrifuged supernatant was then subjected to CPE Western blotting (Supernatant lane of each blot), while the remainder of each sample was applied to parent cells for 1 h. After the cells were washed, those parent cells were subjected to CPE Western blotting (Sup on Parent cell lanes). For comparison, free CPE is shown on each blot. Results shown in panels A and B are representative of three experimental repetitions.

CPE can cause cell lysis and bleb formation in sensitive cells (21, 28), which might release receptor-bound CPE monomer and/or the CH-1 pore complex into supernatants of CPE-treated sensitive cell cultures, possibly allowing those CPE species to be taken up by, and then kill, CPE-insensitive parent cells. This possibility was assessed by evaluating the presence of CPE species in supernatants collected from CPE-treated claudin-4 transfectant cells or CPE-treated Caco-2 cells (Fig. 5A and B). CPE Western blotting did detect considerable amounts of CPE monomer in these supernatants, at least some of which is likely to be free toxin that had not bound to the sensitive cells during CPE treatment. While standard Western blot conditions did not detect CH-1 in supernatants collected from CPE-treated claudin-4 transfectant cells or Caco-2 cells, small amounts of CH-1 were detected on long chemiluminescence exposure of the Western blot (not shown), particularly using supernatants from CPE-treated Caco-2 cells.

**FIG 6:**
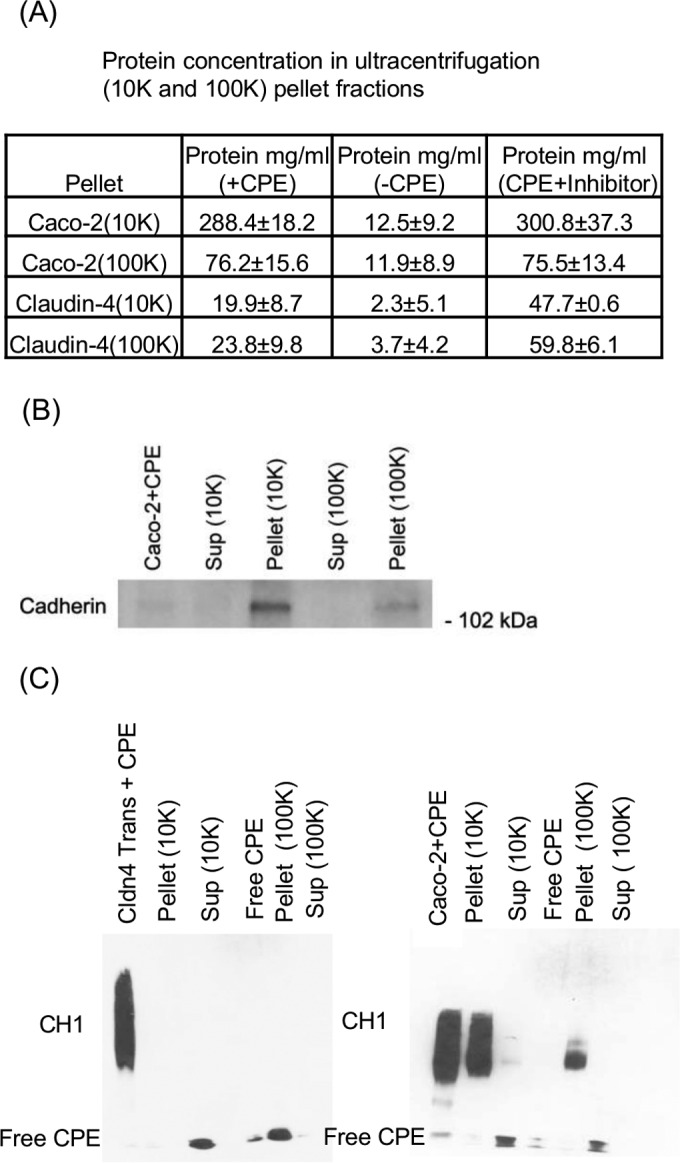
CPE treatment induces sensitive cells to release membrane vesicles containing CPE species. Claudin-4 transfectant cells or Caco-2 cells were incubated for 1 h with 5.0 μg ml^−1^ of CPE or without CPE. The supernatants from those cultures were briefly centrifuged at 2,000 × *g* and then subjected to differential centrifugations at 10,000 × *g* (10K) for 30 min (to collect large extracellular membrane vesicles) and 100,000 × *g* (100J) for 90 min (to collect small extracellular membrane vesicles). The pellets containing membrane vesicles were then analyzed for total protein concentration (A), presence of cadherin as a representative membrane protein (B), or presence of CPE species (C). Panel A also shows the effect on membrane vesicle release when these cells were pretreated with the caspase-3/7 inhibitor Ac-DEVD-CHO prior to this CPE treatment. (C) Results for supernatant fractions from CPE-treated claudin-4 transfectant cells (left) or Caco-2 cells (right). Results in panel A are means of three repetitions (± standard deviation [SD]) while the results in panels B and C are representative of three experimental repetitions.

Differential centrifugation was performed to evaluate whether Caco-2 cells or claudin-4 transfectant cells release extracellular membrane vesicles after CPE treatment. Protein analyses of the obtained pellets detected small (i.e., vesicles pelleted at 100,000 × *g*) and large (vesicles pelleted at 10,000 × *g*) membrane vesicles in supernatants collected from both CPE-treated sensitive cell lines (Fig. 6A), although significantly more membrane vesicles were present in supernatants from CPE-treated Caco-2 cells. The presence of host membrane proteins in pellet fractions containing membrane vesicles released from CPE-treated Caco-2 cells was confirmed by a cadherin Western blot (Fig. 6B); no attempt was made to detect host proteins in CPE-treated claudin-4 transfectant cells due to lower protein levels released by those cells. Compared to supernatants from control Caco-2 or claudin-4 transfectant cells, 10- to 20-fold-more membrane vesicles were present in supernatants collected from sensitive cells after CPE treatment (Fig. 6A).

CPE Western blot analyses were performed to detect CPE in centrifuge fractions collected from CPE-treated sensitive cells (Fig. 6C). Those analyses revealed that CPE monomer was associated with both the large and small membrane vesicles present in supernatants collected from CPE-treated Caco-2 cells and claudin-4 transfectants. In contrast, CH-1 was associated only with vesicles collected from CPE-treated Caco-2 cells. If membrane vesicle-mediated delivery of CH-1 or bound CPE monomer caused the decreased viability in parent cells treated with supernatants from CPE-treated sensitive cells, CPE species should be present in those parent cells. However, Western blot analyses (Fig. 5A and B) detected no CPE in parent cells challenged with supernatants collected from CPE-treated claudin-4 transfectant cells or Caco-2 cells, even using supernatants from sensitive cells challenged with a high (5-μg ml^−1^) CPE dose. The absence of CPE species in the supernatant-treated parent cells was not a CPE Western blot insensitivity issue, since CH-1 complex was readily detected in sensitive cells treated directly with 0.5 μg ml^−1^ of CPE (Fig. 5A and B); that result is informative, since those sensitive cells treated with 0.5 μg ml^−1^ of CPE exhibited cytotoxicity equivalent to that of parent cells challenged with supernatants collected from sensitive cells treated with 5 μg ml^−1^ of CPE.

### Effects of extracellular membrane vesicle depletion on cytotoxic activity in supernatants collected from CPE-treated sensitive cells.

**FIG 7:**
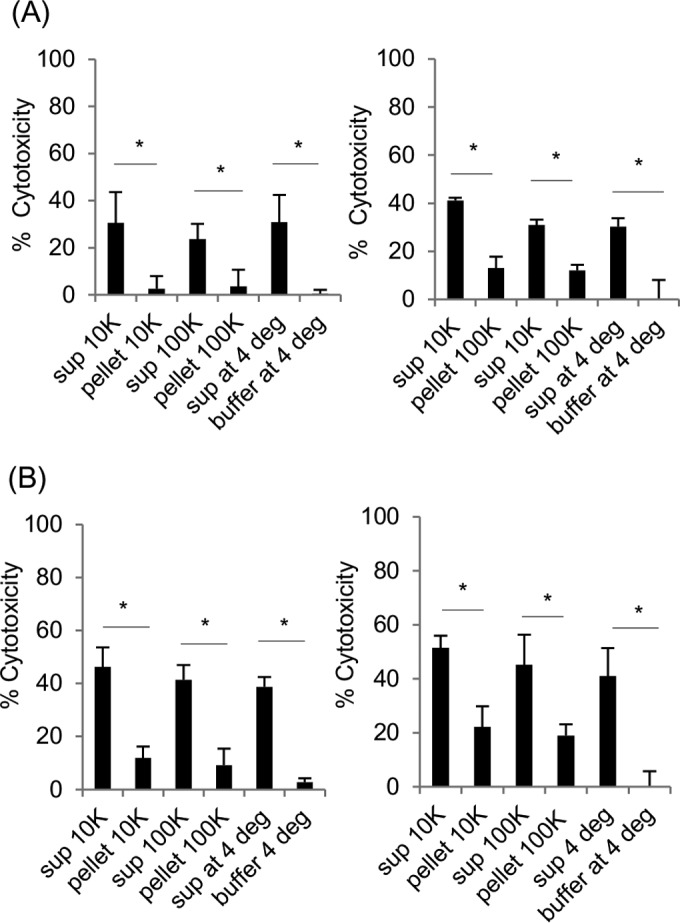
Analysis of the importance of extracellular membrane vesicles for parent cell killing by supernatants from CPE-treated sensitive cells. (A and B) Differential centrifugation fractions of supernatants (sup) from CPE-treated (5.0 μg ml^−1^ of CPE) claudin-4 transfectant cells (A) or Caco-2 cells (B) were applied to parent cells for 1 h at 37°C. These differential centrifugation fractions included (i) pellets containing large (10K) or small (100K) extracellular membrane vesicles that were resuspended back to their original 1× concentrations in crude supernatants or (ii) supernatants from each centrifugation. For comparison, crude supernatants from CPE-treated sensitive cells, or buffer alone, were stored at 4°C (4 deg) for the entire differential centrifugation process and then assayed for cytotoxicity. After treatment with these fractions, LDH (left) or MTT (right) assays were then performed to measure cytotoxicity. Values shown are the means for three experiments. Error bars represent the standard errors of the means. Values that are significantly different (*P* < 0.05) are indicated by a bar and asterisk.

Even if extracellular membrane vesicles were not bringing CPE into parent cells, their presence in supernatants collected from CPE-treated sensitive cells could still contribute to cytotoxicity, since membrane vesicles can deliver toxic factors into host cells (29). To address this possibility, the cytotoxic effects of membrane vesicle-depleted supernatants on parent cells were assessed (Fig. 7A and B). Those analyses demonstrated that supernatants from CPE-treated claudin-4 transfectant cells or Caco-2 cells retain most of their cytotoxic activity after extracellular membrane vesicle depletion. Although significantly less, some cytotoxicity was also detected in parent cells treated with natural concentrations of resuspended membrane vesicles collected from supernatants of those CPE-treated sensitive cells. In contrast, no significant cytotoxicity was detected (data not shown) in parent cells treated with membrane vesicle-depleted supernatants or natural concentrations of membrane vesicles collected from supernatants of control Caco-2 or claudin-4 transfectant cells.

### Characterization of cytotoxic factor activity remaining in extracellular membrane vesicle-depleted supernatants.

Since most cytotoxic activity in crude supernatants collected from CPE-treated sensitive cells remained after depletion of extracellular membrane vesicles by a 100,000 × *g* centrifugation, the cytotoxic factor(s) in those fractions was characterized. These studies (Fig. 4A to D, right panels) showed that the cytotoxic factor(s) present in these membrane vesicle-depleted supernatants was heat labile, with an apparent size of 10 to 30 kDa and sensitive to trypsin. As also noted for crude supernatants, the addition of a serine protease inhibitor significantly reduced the cytotoxic effects of these membrane-depleted supernatants (Fig. 4D, right panel).

### Supernatants collected from CPE-treated sensitive cells induce caspase-3-mediated apoptotic death in parent cells.

**FIG 8:**
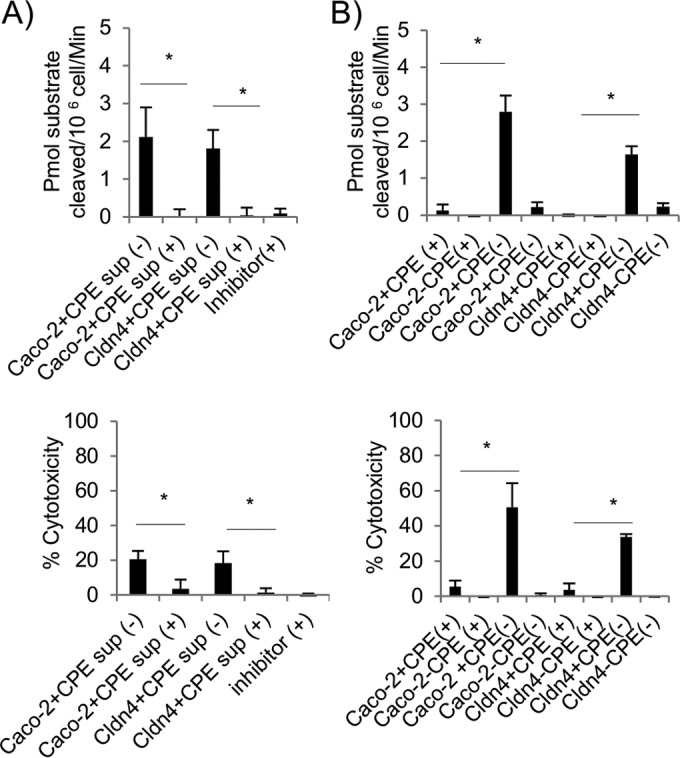
Caspase-3/7 activation in CPE-treated sensitive cells and parent cells treated with supernatants from CPE-treated sensitive cells. (A) Parent cells were treated for 1 h with supernatants collected from Caco-2 cells or claudin-4 transfectant cells that had been treated with 1 μg ml^−1^ of CPE after those cells had been pretreated with the caspase-3/7 inhibitor Ac-DEVD-CHO (+) or had not been pretreated with Ac-DEVD-CHO (−). For a control, a similar experiment was performed where parent cells were treated with supernatant collected from control (no CPE treatment) claudin-4 transfectant cells that had been pretreated with Ac-DEVD-CHO inhibitor only [Inhibitor(+)]. Caspase-3/7 activity (in picomoles of substrate cleaved/10^6^ cells/min) in parent cells is shown in the top graph of panel A, while cytotoxicity (measured by LDH release) in these cells is shown in the bottom graph. (B) Parent cells that had been pretreated for 2 h with the caspase-3/7 inhibitor (+) or had not been pretreated for 2 h with the caspase-3/7 inhibitor (−) before challenge for 1 h with supernatants from Caco-2 cells or claudin-4 transfectants that had been treated for 1 h with 5 μg ml^−1^ of CPE (+CPE) or had not been treated for 1 h with 5 μg ml^−1^ of CPE (-DPE). Caspase-3/7 activity is shown in the top graph in panel B, while cytotoxicity (measured by LDH release) is shown in the bottom graph. Results shown are the means of three repetitions. Error bars depict the standard errors of the means. Values that are significantly different (*P* < 0.05) are indicated by a bar and asterisk.

The final experiments of this study investigated the cell death mechanism triggered when parent cells were treated with supernatants collected from CPE-treated sensitive cells. This analysis first determined (not shown) that treatment with 1 μg ml^−1^ of CPE induces caspase-3 activation in claudin-4 transfectant cells and, as reported previously (23), Caco-2 cells. This caspase-3/7 activation was important for CPE-induced death of these cells, since cytotoxicity was blocked by the caspase-3/7 inhibitor *N*-acetyl-Asp-Glu-Val-Asp-al (Ac-DEVD-CHO) (not shown). The cytotoxic activity in crude supernatants collected from cultures of CPE-treated Caco-2 cells or claudin-4 transfectant cells was dependent upon this caspase 3/7 activation in CPE-sensitive cells. Specifically, pretreating Caco-2 cells or claudin-4 transfectant cells with the caspase 3/7 inhibitor prior to CPE challenge prevented those cells from releasing cytotoxic factors (Fig. 8A), although it did not reduce CPE-induced release of membrane vesicles (Fig. 6A).

Significant caspase-3/7 activity was present in culture supernatants collected from CPE-treated Caco-2 cells or claudin-4 transfectant cells, but not control (no CPE treatment) cells (not shown). However, while direct addition of Ac-DEVD-CHO eliminated caspase 3/7 activity of supernatants collected from CPE-treated Caco-2 cells or claudin-4 transfectant cells, it did not affect the ability of those supernatants to cause cytotoxicity in parent cells (not shown).

The final experiment of this study pretreated parent cells with the caspase-3/7-specific inhibitor. This pretreatment blocked the development of cytotoxicity in those parent cells when they were subsequently treated with supernatants collected from CPE-treated Caco-2 cells or claudin-4 transfectant cells (Fig. 8B).

## DISCUSSION

To our knowledge, the current study provides the first indication that supernatants collected from a pore-forming toxin-treated sensitive cell can kill other cells that are, in pure culture, insensitive to that toxin. This bystander killing effect clearly required the pore-forming activity of CPE since (i) it could be blocked by a CPE-neutralizing monoclonal antibody and (ii) sensitive cells treated with rCPE-D48A, which binds and forms small CPE complex, did not induce release of the cytotoxic factor from these cells. It will be of future interest to evaluate whether a toxin-induced bystander killing phenomenon is common to other pore-forming toxins beyond CPE.

The appearance of membrane blebs on CPE-treated sensitive cells was first noted in 1979 (21, 28). The current study now demonstrates that CPE-treated sensitive cells dying from apoptosis not only form membrane blebs but also release high levels of both small and large membrane vesicles into supernatants. CPE can kill sensitive cells by apoptosis (23, 24; this study), and other studies have shown that membrane blebs carrying cytoplasmic contents can be released from apoptotic cells as apoptotic bodies (30). However, pretreatment of Caco-2 cells or claudin-4 transfectant cells with a caspase-3/7 inhibitor did not affect their release of membrane vesicles upon CPE challenge, strongly suggesting that apoptosis is not required for the release of membrane vesicles from CPE-treated sensitive cells.

The current study also determined that the membrane vesicles released from CPE-treated sensitive cells contain CPE and, for CPE-treated Caco-2 cells, the CH-1 pore. Membrane vesicles released from bacteria can serve as a mechanism for delivering toxins into host cells (31–33), and membrane vesicles released from host cells have been shown to deliver anthrax lethal factor into mammalian cells (34). Therefore, it was conceivable that CPE-containing membrane vesicles might similarly deliver bound CPE monomer or CH-1 into naturally insensitive host cells to create cytotoxic pores. However, no CPE species were detected in parent cells treated with crude supernatants from CPE-treated sensitive cells, even though those supernatants possessed membrane vesicles containing CPE species. Nonetheless, membrane vesicle delivery of CPE species could occur *in vivo*, for example, so further investigation of this potential phenomenon may be warranted.

Although membrane vesicles released from CPE-treated sensitive cells do not introduce CPE into parent cells, they still might deliver toxic host factors into those cells, e.g., the presence of caspase-3, a potent protease, has been detected previously in exosomes (35). Therefore, we tested whether supernatants collected from CPE-treated sensitive cells remained cytotoxic when depleted of membrane vesicles. However, the majority of cytotoxic activity in crude supernatants remained after depletion of membrane vesicles, with membrane vesicles collected from CPE-treated cells causing only a minor increase in parent cell death.

The nature of the cytotoxic activity in crude supernatants and in membrane vesicle-depleted supernatants was further investigated. Characterization studies determined that this factor appears to be a heat-sensitive protein of 10 to 30 kDa. The addition of 200 μg ml^−1^ trypsin inhibitor caused a significant reduction in killing of parent cells by membrane vesicle-depleted supernatants. The addition of twice that amount of trypsin inhibitor did not further reduce cytotoxicity (not shown), suggesting that a cytotoxic factor other than the serine protease is also present in supernatants from CPE-treated sensitive cells.

Serine proteases are involved in apoptosis (36), which is consistent with our observation that the development of apoptosis is important for the release of cytotoxic factors into the supernatants of CPE-treated sensitive cells. Whether the serine protease present in supernatants from CPE-treated sensitive cells is directly responsible for parent cell killing or instead activates another factor will require further work to determine. Barely detectable (equivalent to <1 μg/ml of trypsin) protease activity was detected in membrane vesicle-depleted supernatants, and most of this activity was sensitive to trypsin inhibitor (data not shown). Those low levels of serine protease activity in supernatants from CPE-treated cells, coupled with the observation that there appear to be more than one cytotoxic factor in these supernatants, will make future analyses of the cytotoxic factors by conventional biochemical approaches challenging.

The presence of some serine protease activity in supernatants is not surprising given that a number of other host proteins, such as LDH and caspase-3, were also detected in supernatants collected from CPE-treated sensitive cells. However, caspase-3 activity itself was clearly not responsible for the cytotoxic activity in supernatants from CPE-treated sensitive cells, since inhibition of caspase-3/7 activity in supernatants collected from CPE-treated sensitive cells did not reduce the cytotoxic activity of those supernatants.

The discovery in this study that CPE-sensitive cells release a cytotoxic factor(s) to damage other cells that is inherently insensitive to the direct effects of this toxin may have biomedical significance. Since the CPE quantities used in this study have pathophysiologic relevance (37), this bystander killing effect could contribute to CPE-mediated gastrointestinal (GI) disease. In the GI tract, CPE damages sensitive cells after binding to claudin receptors on enterocytes (37). However, the availability of claudin CPE receptors varies considerably among different intestinal segments, along the crypt to villus/surface cell axis in the intestines, by subcellular distribution and with age (38–40). Therefore, during GI disease, it is likely that only some cells respond directly to CPE, as supported by immunolocalization experiments showing CPE binding predominantly to villus tips in rabbit small intestine, where CPE damage begins (37). However, in the GI tract, CPE-sensitive cells are likely in the proximity of CPE-insensitive cells. Our current work could suggest that CPE induces release of cytotoxic factors from those CPE-sensitive cells, which could damage nearby CPE-insensitive cells and contribute to pathology.

In addition, epidemiological evidence and animal model studies strongly suggest that, when preexisting conditions exist that block the development of diarrhea (such as severe constipation or fecal impaction due to medication side effects), CPE can cause enterotoxemia (41, 42). In this situation, the enterotoxin can be absorbed from the intestines into the circulation system, where it then damages organs such as the kidneys and liver. Those organs are also a complex mix of CPE-sensitive and CPE-insensitive cells, where release of cytotoxic factors from CPE-sensitive cells might contribute to pathology by inducing damage to nearby CPE-insensitive cells. These possibilities suggest a need for future studies to examine whether CPE can affect naturally insensitive cells in the intestines or other organs.

Last, there is currently significant interest in translational use of CPE for cancer therapy and diagnosis or drug delivery. It is conceivable that the release of cytotoxic factors from CPE-treated sensitive cells could complicate some of these translational efforts when insensitive cells are in close proximity to CPE-sensitive target cells.

## MATERIALS AND METHODS

### Materials.

Native *Clostridium perfringens* enterotoxin (CPE) was purified to homogeneity as described previously (43). Rabbit polyclonal antibody was raised against purified CPE as described previously (44). CPE-neutralizing monoclonal antibody 3C9 and nonneutralizing CPE monoclonal antibody 10G6 were prepared previously (45) and purified by standard techniques (45). *E. coli* transformants producing recombinant CPE (rCPE) or the rCPE-D48A variant (rCPE with D48A change) were enriched to near homogeneity by Talon resin chromatography (Qiagen) as described previously (26). Protein concentrations were determined for differential centrifugation fractions using the Pierce 660 nm protein assay (Thermo Fisher) and for other samples using the Pierce BCA protein assay kit (Thermo Fisher). Protease activity in supernatants was measured using the Pierce protease assay kit (Thermo Fisher).

### Treatment of cell cultures with CPE species.

Rat1-R12 parental fibroblasts do not naturally express claudins (17, 46, 47) and are referred to as parent cells. Previously prepared (48) Rat1-R12 fibroblast transfectant cells that stably express claudin-4 are referred to as claudin-4 transfectant cells. Authenticated Caco-2 cells were purchased from ATCC. Unless otherwise specified, cell cultures were treated with Hanks balanced salt solution (HBSS) containing 0.5 or 5 μg ml^−1^ of a CPE species (CPE, rCPE, or rCPE-D48A) for 1 h at 37°C.

### Microscopy.

Washed parent cells were suspended at 3 × 10^6^ cells/ml in phosphate-buffered saline (PBS) at room temperature (RT) and then stained with 1 μM CFSE [5- (and 6-)-carboxyfluorescein diacetate succinimidyl ester (eBioscience)] for 10 min at RT in the dark. The reaction was stopped by adding 4 to 5 volumes of cold complete minimum essential medium (MEM), followed by incubation on ice for 5 min. After three washes with complete MEM, pure cultures of those stained cells, or unstained claudin-4 transfectant cells, were cultured to confluence in an eight-well microscopy slide (Lab-Tek) coated with Vitrogen plating medium MEM clear (Life Technology), 1 mg ml^−1^ fibronectin (Fisher), 7.5% bovine serum albumin (BSA) (Life Technology), and 3 mg ml^−1^ bovine collagen (Fisher). To prepare mixed cultures of unstained claudin-4 transfectant cells together with CFSE-stained parent cells, each cell type was inoculated at a 1:1 ratio and cultured to confluence on a Vitrogen-coated microscopy chamber slide. The pure or mixed cell cultures were treated with CPE, washed twice with PBS, and stained with the fixable viability dye eFluor 450 (eBioscience) at a 1:10,000 dilution for 30 min at 4°C in the dark. After two washes with PBS, the cells were fixed with 4% paraformaldehyde for 20 min in the dark at RT. The fixed cells were washed twice with PBS and then stained with Hoechst 33342 (Invitrogen) at a 1:10,000 dilution for 10 min at RT. Microscopy was performed using an Olympus confocal laser scanning biological microscope (FluoView FV1000) with FV10-ASW (version 1.4) software. All pictures were taken at a magnification of 200×. Quantification of cell viability (eFluor 450-stained cells) or CFSE staining was performed by counting six microscopic fields for each treatment condition in each experiment, with this analysis repeated in three independent experiments.

### CPE Western blot analysis of CH-1 formation.

CPE-treated Caco-2 cells or claudin-4 transfectant cells were washed with HBSS, suspended in PBS, and centrifuged at 2,000 × *g* for 3 min. The cell pellet was then resuspended in 50 μl of radioimmunoprecipitation assay (RIPA) buffer and processed for CPE Western immunoblotting as described previously (19). Similarly, supernatants from CPE-treated claudin-4 transfectants or Caco-2 cell cultures were directly analyzed by CPE Western blotting or applied to parent cells for 1 h at 37°C. After the cells were washed, the supernatant-treated parent cells were analyzed by CPE Western blotting (19).

### Analysis of culture supernatant effects on cytotoxicity.

CPE species-treated confluent parent cells, claudin-4 transfectant cells, or Caco-2 cells were collected by centrifugation at 2,000 × *g* for 3 min, and supernatants from those centrifugations were applied to fresh confluent parent cell cultures for 1 h at 37°C. Cytotoxicity in these cultures was measured by microscopy (as described above) or by using an MTT assay kit (CellTiter 96 Aqueous one-solution cell proliferation assay; Promega) or LDH release assay (Roche) per the manufacturer’s instructions. LDH release specifically induced by supernatant treatment of parent cells was calculated after subtracting the LDH already present in the supernatants due to its release from CPE-treated Caco-2 cells or claudin-4 transfectants.

### Analysis of release of cytotoxic factor(s) by CPE-treated sensitive cells.

CPE-treated claudin-4 transfectant cells were centrifuged for 3 min at 2,000 × *g*. The resultant supernatants were processed to evaluate the protease sensitivity of their cytotoxic activity after treatment with or without 100 μg ml^−1^ of trypsin (Sigma) for 1 h at 37°C. Trypsin inhibitor (200 or 400 μg ml^−1^; Sigma) in HBSS was added, and after 30 min at RT, the samples were applied to confluent parent cells for 1 h at 37°C. An MTT assay was then performed to determine cytotoxicity. The size of cytotoxic factor(s) in the supernatants was evaluated by centrifuging the supernatants on 10-kDa- or 30-kDa-cutoff Amicon filters (Millipore). The flowthrough and retained fractions were separately applied to confluent cultures of the parent cells, and an MTT assay was performed. The heat sensitivity of cytotoxic factor(s) was evaluated by heating the supernatants at 70°C for 10 min. Heated and unheated supernatants were then separately applied to confluent cultures of the parent cells, and an MTT cytotoxicity assay was performed.

Trypsin sensitivity, trypsin inhibitor sensitivity, heat sensitivity, size of cytotoxic factor(s), and protease activity in supernatants from CPE-treated claudin-4 transfectants that were depleted of membrane vesicles by ultracentrifugation at 100,000 × *g* for 90 min were examined. An aliquot of those supernatants was also ultrafiltered through 30-kDa-cutoff Amicon filters.

### Antibody neutralization of CPE activity.

CPE (1 μg ml^−1^) alone or in the presence of 10 μg ml^−1^ of MAb3C9 or MAb10G6 was preincubated in HBSS at 37°C for 1 h. For controls, the same amounts of those monoclonal antibodies were similarly preincubated in HBSS without CPE. After preincubation, samples were added to claudin-4 transfectants for 1 h at 37°C. Culture supernatants were then collected by centrifugation at 2,000 × *g* for 3 min, and supernatants from those centrifugations were applied to parent cells for 1 h at 37°C, followed by cytotoxicity assay using an MTT assay.

### Differential centrifugation fractionation of supernatants from CPE-treated sensitive cells.

CPE-treated claudin-4 transfectant or Caco-2 cell cultures were centrifuged at 2, 000 × *g* for 3 min, and those supernatants were subjected to sequential centrifugation (30 min at 10,000 × *g* to collect large membrane vesicles and then 90 min at 100,000 × *g* to collect small membrane vesicles [49, 50]). Pelleted membrane vesicles resuspended back to natural (1×) concentrations, or supernatant fractions from centrifugations, were applied to parent cells for MTT cytotoxicity testing or Western blotted with a cadherin antibody (Cell Signaling Technology, Inc.) to validate vesicle depletion from supernatants. For cadherin Western blots, equal amounts of protein were separated by SDS-PAGE on Tris gels (Bio-Rad) and transferred to a polyvinylidene difluoride (PVDF) membrane.

### Caspase-3/7 activity in parent cells challenged with supernatants from CPE-treated sensitive cells.

Claudin-4 transfectant cells or Caco-2 cells were preincubated for 2 h at 37°C in HBSS with or without 50 μM caspase-3/7 inhibitor *N*-acetyl-Asp-Glu-Val-Asp-al (Ac-DEVD-CHO) (Sigma). After two washes with HBSS, 1 μg CPE ml^−1^ was added in the presence or absence of 50 μM Ac-DEVD-CHO. After 1 h at 37°C, the CPE-treated cells were washed twice with HBSS and collected by centrifugation. The cell pellets were suspended in lysis buffer {50 mM HEPES (pH 7.4), 0.1% 3-[(3-cholamidopropyl)-dimethylammonio]-1-propanesulfonate (CHAPS), 1 mM dithiothreitol, 0.1 mM EDTA} for 5 min on ice. Those lysates were centrifuged at 10,000 × *g* for 10 min at 4°C. Supernatants were tested for caspase-3/7 activity using 2 mM caspase-3/7 colorimetric substrate (Ac-DEVD-pNA [*p*-nitroanilide]; Sigma) dissolved in assay buffer [50 mM HEPES (pH 7.4), 100 mM NaCl, 0.1% CHAPS, 10 mM dithiothreitol, 1 mM EDTA, 10% glycerol]. A 10-μl aliquot of each cell lysate (as prepared above), 10 μl of caspase substrate solution, and assay buffer were combined in a total 100-μl volume and incubated at 37°C for 4 h. Release of *p*-nitroaniline from the caspase-3 substrate was detected by measuring optical density at 405 nm (OD_405_) using a Bio-Rad plate reader. The amount of substrate cleaved was calculated according to the manufacturer’s instructions. Supernatants from the CPE-treated Caco-2 cells and claudin-4 transfectants were also directly assayed for caspase-3/7 and LDH activity before being applied to parent cells.

Parent cells were also preincubated for 1 h at 37°C in HBSS with a 50 μM concentration of Ac-DEVD-CHO before 1-h challenge with supernatants collected from sensitive cells that had been treated for 1 h with 5 μg ml^−1^ of CPE. Those supernatant-treated parent cells were assessed for LDH release, and after they were washed, caspase-3/7 activity assay as described above.

### Statistical analyses.

Multiple comparisons used one-way analysis of variance (ANOVA) with the Dunnett’s multiple comparison test. For pairwise data comparisons, Student’s *t* test was performed.

## References

[1] Czeczulin JR, Hanna PC, McClane BA 1993 Cloning, nucleotide sequencing, and expression of the *Clostridium perfringens* enterotoxin gene in *Escherichia coli*. Infect Immun 61:3429–3439. 8335373 10.1128/iai.61.8.3429-3439.1993 PMC281020

[2] Van Itallie CM, Betts L, Smedley JG III, McClane BA, Anderson JM 2008 Structure of the claudin-binding domain of *Clostridium perfringens* enterotoxin. J Biol Chem 283:268–274. doi:10.1074/jbc.M708066200. 17977833

[3] Kitadokoro K, Nishimura K, Kamitani S, Fukui-Miyazaki A, Toshima H, Abe H, Kamata Y, Sugita-Konishi Y, Yamamoto S, Karatani H, Horiguchi Y 2011 Crystal structure of *Clostridium perfringens* enterotoxin displays features of beta-pore-forming toxins. J Biol Chem 286:19549–19555. doi:10.1074/jbc.M111.228478. 21489981 PMC3103334

[4] Briggs DC, Naylor CE, Smedley JG III, Lukoyanova N, Robertson S, Moss DS, McClane BA, Basak AK 2011 Structure of the food-poisoning *Clostridium perfringens* enterotoxin reveals similarity to the aerolysin-like pore-forming toxins. J Mol Biol 413:138–149. doi:10.1016/j.jmb.2011.07.066. 21839091 PMC3235586

[5] McClane BA, Robertson SL, Li J 2013 Clostridium perfringens, p 465–489. *In* Doyle MP, Buchanan RL (ed), Food microbiology: fundamentals and frontiers, 4th ed. ASM Press, Washington, DC.

[6] Sarker MR, Carman RJ, McClane BA 1999 Inactivation of the gene (*cpe*) encoding *Clostridium perfringens* enterotoxin eliminates the ability of two *cpe*-positive *C. perfringens* type A human gastrointestinal disease isolates to affect rabbit ileal loops. Mol Microbiol 33:946–958. doi:10.1046/j.1365-2958.1999.01534.x. 10476029

[7] Scallan E, Hoekstra RM, Angulo FJ, Tauxe RV, Widdowson MA, Roy SL, Jones JL, Griffin PM 2011 Foodborne illness acquired in the United States−major pathogens. Emerg Infect Dis 17:7–15. doi:10.3201/eid1701.091101p1. 21192848 PMC3375761

[8] McClane BA, Uzal FA, Miyakawa MF, Lyerly D, Wilkins TD 2006 The enterotoxic clostridia, p 688–752. *In* Dworkin M, Falkow S, Rosenburg E, Schleifer H, Stackebrandt E (ed), The prokaryotes: a handbook on the biology of bacteria, 3rd ed. Springer, New York, NY.

[9] Ma M, Gurjar A, Theoret JR, Garcia JP, Beingesser J, Freedman JC, Fisher DJ, McClane BA, Uzal FA 2014 Synergistic effects of *Clostridium perfringens* enterotoxin and beta toxin in rabbit small intestinal loops. Infect Immun 82:2958–2970. doi:10.1128/IAI.01848-14. 24778117 PMC4097624

[10] Krause G, Protze J, Piontek J 2015 Assembly and function of claudins: structure-function relationships based on homology models and crystal structures. Semin Cell Dev Biol 42:3–12. doi:10.1016/j.semcdb.2015.04.010. 25957516

[11] Katahira J, Sugiyama H, Inoue N, Horiguchi Y, Matsuda M, Sugimoto N 1997 *Clostridium perfringens* enterotoxin utilizes two structurally related membrane proteins as functional receptors *in* *vivo*. J Biol Chem 272:26652–26658. doi:10.1074/jbc.272.42.26652. 9334247

[12] Shrestha A, McClane BA 2013 Human claudin-8 and -14 are receptors capable of conveying the cytotoxic effects of *Clostridium perfringens* enterotoxin. mBio 4:e00594-12. doi:10.1128/mBio.00594-12. 23322640 PMC3551551

[13] Fujita K, Katahira J, Horiguchi Y, Sonoda N, Furuse M, Tsukita S 2000 *Clostridium perfringens* enterotoxin binds to the second extracellular loop of claudin-3, a tight junction integral membrane protein. FEBS Lett 476:258–261. doi:10.1016/S0014-5793(00)01744-0. 10913624

[14] Mitchell LA, Koval M 2010 Specificity of interaction between *Clostridium perfringens* enterotoxin and claudin-family tight junction proteins. Toxins 2:1595–1611. doi:10.3390/toxins2071595. 22069652 PMC3153273

[15] Shrestha A, Uzal FA, McClane BA 2016 The interaction of *Clostridium perfringens* enterotoxin with receptor claudins. Anaerobe 41:18–26. doi:10.1016/j.anaerobe.2016.04.011. 27090847 PMC5050067

[16] Wieckowski EU, Wnek AP, McClane BA 1994 Evidence that an approximately 50-kDa mammalian plasma membrane protein with receptor-like properties mediates the amphiphilicity of specifically bound *Clostridium perfringens* enterotoxin. J Biol Chem 269:10838–10848. 8144671

[17] Singh U, Van Itallie CM, Mitic LL, Anderson JM, McClane BA 2000 CaCo-2 cells treated with *Clostridium perfringens* enterotoxin form multiple large complex species, one of which contains the tight junction protein occludin. J Biol Chem 275:18407–18417. doi:10.1074/jbc.M001530200. 10749869

[18] Smedley JG III, Uzal FA, McClane BA 2007 Identification of a prepore large-complex stage in the mechanism of action of *Clostridium perfringens* enterotoxin. Infect Immun 75:2381–2390. doi:10.1128/IAI.01737-06. 17307943 PMC1865780

[19] Robertson SL, Smedley JG III, Singh U, Chakrabarti G, Van Itallie CM, Anderson JM, McClane BA 2007 Compositional and stoichiometric analysis of *Clostridium perfringens* enterotoxin complexes in Caco-2 cells and claudin 4 fibroblast transfectants. Cell Microbiol 9:2734–2755. doi:10.1111/j.1462-5822.2007.00994.x. 17587331

[20] Chen J, Theoret JR, Shrestha A, Smedley JG III, McClane BA 2012 Cysteine-scanning mutagenesis supports the importance of *Clostridium perfringens* enterotoxin amino acids 80 to 106 for membrane insertion and pore formation. Infect Immun 80:4078–4088. doi:10.1128/IAI.00069-12. 22966051 PMC3497400

[21] Matsuda M, Sugimoto N 1979 Calcium-independent and dependent steps in action of *Clostridium perfringens* enterotoxin on HeLa and Vero cells. Biochem Biophys Res Commun 91:629–636. doi:10.1016/0006-291X(79)91568-7. 229852

[22] McClane BA, Wnek AP, Hulkower KI, Hanna PC 1988 Divalent cation involvement in the action of *Clostridium perfringens* type A enterotoxin. Early events in enterotoxin action are divalent cation-independent. J Biol Chem 263:2423–2435. 3123494

[23] Chakrabarti G, Zhou X, McClane BA 2003 Death pathways activated in CaCo-2 cells by *Clostridium perfringens* enterotoxin. Infect Immun 71:4260–4270. doi:10.1128/IAI.71.8.4260-4270.2003. 12874301 PMC166005

[24] Chakrabarti G, McClane BA 2005 The importance of calcium influx, calpain and calmodulin for the activation of CaCo-2 cell death pathways by *Clostridium perfringens* enterotoxin. Cell Microbiol 7:129–146. doi:10.1111/j.1462-5822.2004.00442.x. 15617529

[25] Sherman S, Klein E, McClane BA 1994 *Clostridium perfringens* type A enterotoxin induces concurrent development of tissue damage and fluid accumulation in the rabbit ileum. J Diarrhoeal Dis Res 12:200–207. 7868827

[26] Smedley JG III, McClane BA 2004 Fine mapping of the N-terminal cytotoxicity region of *Clostridium perfringens* enterotoxin by site-directed mutagenesis. Infect Immun 72:6914–6923. doi:10.1128/IAI.72.12.6914-6923.2004. 15557612 PMC529159

[27] McDonel JL 1974 *In vivo* effects of *Clostridium perfringens* enteropathogenic factors on the rat ileum. Infect Immun 10:1156–1162. 16558104 10.1128/iai.10.5.1156-1162.1974 PMC423076

[28] McClane BA, McDonel JL 1979 The effects of *Clostridium perfringens* enterotoxin on morphology, viability, and macromolecular synthesis in Vero cells. J Cell Physiol 99:191–200. doi:10.1002/jcp.1040990205. 222780

[29] De Toro J, Herschlik L, Waldner C, Mongini C 2015 Emerging roles of exosomes in normal and pathological conditions: new insights for diagnosis and therapeutic applications. Front Immunol 6:203. doi:10.3389/fimmu.2015.00203. 25999947 PMC4418172

[30] Poon IK, Lucas CD, Rossi AG, Ravichandran KS 2014 Apoptotic cell clearance: basic biology and therapeutic potential. Nat Rev Immunol 14:166–180. doi:10.1038/nri3607. 24481336 PMC4040260

[31] Bomberger JM, Maceachran DP, Coutermarsh BA, Ye S, O’Toole GA, Stanton BA 2009 Long-distance delivery of bacterial virulence factors by *Pseudomonas aeruginosa* outer membrane vesicles. PLoS Pathog 5:e1000382. doi:10.1371/journal.ppat.1000382. 19360133 PMC2661024

[32] Kesty NC, Mason KM, Reedy M, Miller SE, Kuehn MJ 2004 Enterotoxigenic Escherichia coli vesicles target toxin delivery into mammalian cells. EMBO J 23:4538–4549. doi:10.1038/sj.emboj.7600471. 15549136 PMC533055

[33] Rivera J, Cordero RJ, Nakouzi AS, Frases S, Nicola A, Casadevall A 2010 *Bacillus anthracis* produces membrane-derived vesicles containing biologically active toxins. Proc Natl Acad Sci U S A 107:19002–19007. doi:10.1073/pnas.1008843107. 20956325 PMC2973860

[34] Abrami L, Brandi L, Moayeri M, Brown MJ, Krantz BA, Leppla SH, van der Goot FG 2013 Hijacking multivesicular bodies enables long-term and exosome-mediated long-distance action of anthrax toxin. Cell Rep 5:986–996. doi:10.1016/j.celrep.2013.10.019. 24239351 PMC3866279

[35] Böing AN, Stap J, Hau CM, Afink GB, Ris-Stalpers C, Reits EA, Sturk A, van Noorden CJ, Nieuwland R 2013 Active caspase-3 is removed from cells by release of caspase-3-enriched vesicles. Biochim Biophys Acta 1833:1844–1852. doi:10.1016/j.bbamcr.2013.03.013. 23531593

[36] Moffitt KL, Martin SL, Walker B 2007 The emerging role of serine proteases in apoptosis. Biochem Soc Trans 35:559–560. doi:10.1042/BST0350559. 17511651

[37] Smedley JG III, Saputo J, Parker JC, Fernandez-Miyakawa ME, Robertson SL, McClane BA, Uzal FA 2008 Noncytotoxic *Clostridium perfringens* enterotoxin (CPE) variants localize CPE intestinal binding and demonstrate a relationship between CPE-induced cytotoxicity and enterotoxicity. Infect Immun 76:3793–3800. doi:10.1128/IAI.00460-08. 18505809 PMC2493238

[38] Fujita H, Chiba H, Yokozaki H, Sakai N, Sugimoto K, Wada T, Kojima T, Yamashita T, Sawada N 2006 Differential expression and subcellular localization of claudin-7, -8, -12, -13, and -15 along the mouse intestine. J Histochem Cytochem 54:933–944. doi:10.1369/jhc.6A6944.2006. 16651389

[39] Holmes JL, Van Itallie CM, Rasmussen JE, Anderson JM 2006 Claudin profiling in the mouse during postnatal intestinal development and along the gastrointestinal tract reveals complex expression patterns. Gene Expr Patterns 6:581–588. doi:10.1016/j.modgep.2005.12.001. 16458081

[40] Lameris AL, Huybers S, Kaukinen K, Mäkelä TH, Bindels RJ, Hoenderop JG, Nevalainen PI 2013 Expression profiling of claudins in the human gastrointestinal tract in health and during inflammatory bowel disease. Scand J Gastroenterol 48:58–69. doi:10.3109/00365521.2012.741616. 23205909

[41] Bos J, Smithee L, McClane B, Distefano RF, Uzal F, Songer JG, Mallonee S, Crutcher JM 2005 Fatal necrotizing colitis following a foodborne outbreak of enterotoxigenic *Clostridium perfringens* type A infection. Clin Infect Dis 40:e78–e83. doi:10.1086/429829. 15844055

[42] Caserta JA, Robertson SL, Saputo J, Shrestha A, McClane BA, Uzal FA 2011 Development and application of a mouse intestinal loop model to study the in vivo action of *Clostridium perfringens* enterotoxin. Infect Immun 79:3020–3027. doi:10.1128/IAI.01342-10. 21628512 PMC3147562

[43] McDonel JL, McClane BA 1988 Production, purification, and assay of *Clostridium perfringens* enterotoxin. Methods Enzymol 165:94–103. 2906731 10.1016/s0076-6879(88)65018-x

[44] McClane BA, Strouse RJ 1984 Rapid detection of *Clostridium perfringens* type A enterotoxin by enzyme-linked immunosorbent assay. J Clin Microbiol 19:112–115. 6321542 10.1128/jcm.19.2.112-115.1984 PMC270997

[45] Wnek AP, Strouse RJ, McClane BA 1985 Production and characterization of monoclonal antibodies against *Clostridium perfringens* type A enterotoxin. Infect Immun 50:442–448. 2865210 10.1128/iai.50.2.442-448.1985 PMC261972

[46] Van Itallie CM, Anderson JM 1997 Occludin confers adhesiveness when expressed in fibroblasts. J Cell Sci 110:1113–1121. 9175707 10.1242/jcs.110.9.1113

[47] Singh U, Mitic LL, Wieckowski EU, Anderson JM, McClane BA 2001 Comparative biochemical and immunocytochemical studies reveal differences in the effects of *Clostridium perfringens* enterotoxin on polarized CaCo-2 cells versus Vero cells. J Biol Chem 276:33402–33412. doi:10.1074/jbc.M104200200. 11445574

[48] Robertson SL, Smedley JG III, McClane BA 2010 Identification of a claudin-4 residue important for mediating the host cell binding and action of *Clostridium perfringens* enterotoxin. Infect Immun 78:505–517. doi:10.1128/IAI.00778-09. 19884339 PMC2798200

[49] Bobrie A, Colombo M, Krumeich S, Raposo G, Théry C 2012 Diverse subpopulations of vesicles secreted by different intracellular mechanisms are present in exosome preparations obtained by differential ultracentrifugation. J Extracell Vesicles 1:18397. doi:10.3402/jev.v1i0.18397. PMC3760636 24009879

[50] Liu S, Hossinger A, Hofmann JP, Denner P, Vorberg IM 2016 Horizontal transmission of cytosolic sup35 prions by extracellular vesicles. mBio 7:e00915-16. doi:10.1128/mBio.00915-16. 27406566 PMC4958257

